# Virtual reality vs. physical models in surgical skills training. An update of the evidence

**DOI:** 10.1097/MOU.0000000000001145

**Published:** 2023-11-15

**Authors:** Baldev Chahal, Abdullatif Aydin, Kamran Ahmed

**Affiliations:** a MRC Centre for Transplantation, Guy's Hospital, King's College London; b Department of Urology, King's College Hospital NHS Foundation Trust, London, UK; c Khalifa University; d Department of Urology, Sheikh Khalifa Medical City, Abu Dhabi, United Arab Emirates

**Keywords:** education, simulation, skills, validity

## Abstract

**Purpose of review:**

Simulation is a key component of surgical training, enabling trainees to develop their skills in a safe environment. With simulators broadly grouped into physical models and virtual-reality (VR) simulators, it is important to evaluate the comparative effectiveness of the simulator types in terms of validity as well as cost. The review aims to compare the benefits and drawbacks of novel VR and physical simulators within the broader themes of endourology, laparoscopic and robotic operations, and other urological procedures.

**Recent findings:**

Key benefits of bench models include their comparatively lower cost, easy access and provision of haptic feedback, whereas VR simulators are generally self-sufficient, reusable and enable skills of haemostasis to be practised. The advent of perfused 3D printed simulators across a range of urological procedures may replace cadavers as the traditional gold-standard simulation modality.

**Summary:**

Although possessing differing strengths and downsides, VR and physical simulators when used together can have an additive effect due to skill transferability across the platforms. Further comparative studies are required to directly quantify the differences between physical models and VR simulators in terms of performance metrics and cost-effectiveness. There is lack of validated VR simulators for open and reconstructive procedures.

## INTRODUCTION

Simulation is an integral part of surgical training enabling trainees to hone their technical skills away from actual patients until they achieve a safe level of competency [1]. This is particularly relevant in urology with multiple procedures possessing lengthy learning curves such as percutaneous nephrolithotomy (PCNL) which can require over 100 cases for proficiency [2]. Simulation exists in two principal forms; physical models and virtual-reality (VR) simulators. Physical models include synthetic ‘dry-lab’ bench models and ‘wet-lab’ cadaver and animal models [3]. The effectiveness of a simulator as an educational tool is evaluated through validation studies, with face, content and construct validity widely considered as the minimum criteria a simulator should meet in order to justify its widespread use [4]. Face validity refers to user opinion of a simulator's realism while content validity is based on expert opinion as to how well the simulator's content reflects the necessary skills and knowledge required. Construct validity is demonstrated when nonexperts are outperformed by experts in standardised tasks [5]. Other important considerations in evaluating simulators include their cost-effectiveness and availability. Leading on from this, although comparative studies have concluded that bench and VR models have equivalent training efficacy [6], bench models and VR models have different financial cost profiles which need to be factored in alongside their respective validities. This review aims to provide an update of the evidence, comparing the benefits and drawbacks of physical and VR urological simulators thematically, categorised into simulators for endourology, laparoscopic and robotic procedures, and other urological procedures. 

**Box 1:**
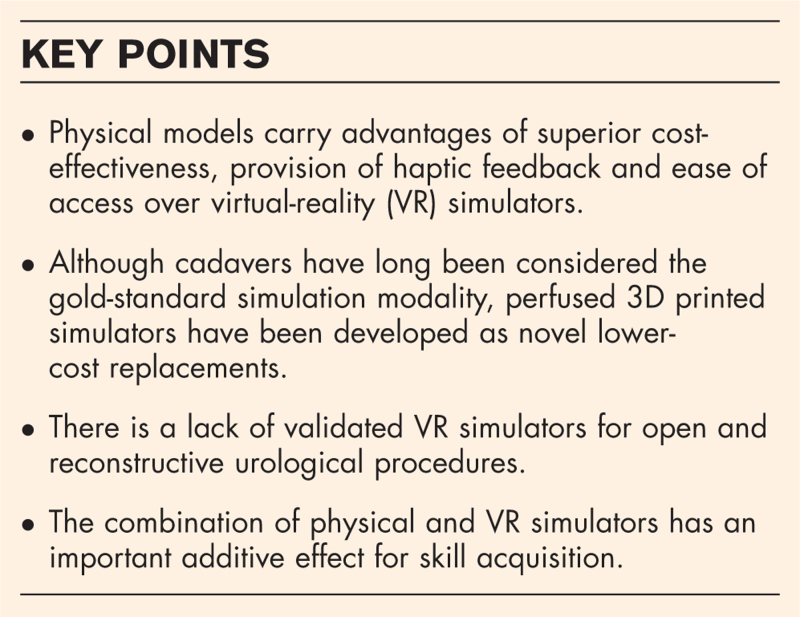
no caption available

## ENDOUROLOGY

Endourological procedures include ureteroscopy, cystoscopy and transurethral resections of the bladder and prostate. The SIMULATE international prospective trial [7] examined the transferability of ureteroscopy skills gained using the URO Mentor VR simulator, Advanced Scope Trainer, Uro-Scopic Trainer and fresh frozen cadavers (FFCs) to the operating room. Regarding content validity, the URO Mentor VR simulator was rated inferiorly to the other modalities with its outdated technology and high cost, with its role purported to be best suited to familiarisation with instruments and cognitive preparation. The dry-lab models were rated highly with their usefulness attributed to their provision of tangible feedback and use of real instruments with irrigation, costing significantly less than the VR simulator or FFCs. The study raised the issue of cost-effectiveness of FFCs, as there was no statistically significant difference in performances between FFC and non-FFC cohorts in the operative setting with participants also reporting difficulty in ureteric navigation. The authors recommend that the effectiveness of FFCs lies in later stages of training with multiple procedures practised on them to enhance cost-effectiveness. Assessment-wise, VR carries the benefits of providing performance feedback whereas the physical models rely upon the presence of live or recorded observers.

3D model simulators have been recently developed in the field of cystoscopy such as the ‘FlexBlad’ [8] and ‘BladCap’ [9]. The ‘FlexBlad’ is a soft urinary bladder phantom [8], displaying adjustable physiological compliance as well as realistic vasculature. The model demonstrated content, face and construct validity, with its particular strengths including its realism as well as possessing reconfigurable tumours to enable trainees to better understand anatomical variations; a feature more typically associated with VR simulators. For transurethral resections of bladder tumours (TURBT) specifically, a high-fidelity VR simulator [10] has been demonstrated to have somewhat acceptable levels of face and content validity but failed to exhibit construct validity. The authors suggest the poor simulation of depth of resection prevented a level of difficulty sufficient to differentiate between experienced and novice surgeons; this is in contrast to the bladder phantom models which better simulate this procedural aspect. One comparative study [11] suggested an additive effect of VR and low-fidelity dry-lab models in enhancing skill development for transurethral resections. The VR TURPMentor and physical Samed simulator were utilised by trainees, achieving high ratings and improving confidence. The TURPMentor's strengths over the physical simulator included the simulation of bleeding, haemostasis and anatomical variation but its main drawback was the lack of haptic feedback. In contrast, the physical simulator provided realistic haptic feedback but whereas the VR simulator is self-sufficient, the Samed simulator has additional costs associated with the use of disposables and requirement to have a technician present for the maintenance of the simulator.

The European Section of Uro-Technology (ESUT) undertook a systematic review [12^▪▪^] examining simulation training in transurethral resection and laser vaporisation procedures of the prostate. The review findings pointed towards VR simulators uniquely possessing data capture and performance evaluation features but that alongside their typically poor haptic feedback, their simulation of bleeding is generally poor for TURP and laser vaporisation. Physical simulators such as bench-top tissue and food-based models also inadequately simulate bleeding for these procedures and carry hygiene risks, but are lower cost and beneficial in that they use real instruments. In the context of laser-based procedures, cadavers demonstrate content validity for holmium laser AEEP training [13] with superior anatomical fidelity to VR simulators and other physical simulators. Highly-rated aspects of cadavers included their provision of anatomic landmarks and realistic simulation of creating the layer between the adenoma and prostate capsule. Alongside the well described drawbacks of cost and availability, cadavers were rated as inadequate specifically for simulation of the laser-tissue reaction. In contrast, as described in another ESUT review [14], VR simulators such as the UroSim HoLEP simulator score highly for simulation of laser-tissue reaction, irrigation and bubbles whilst also providing a degree of haptic feedback. Disadvantages of these VR simulators such as the lack of real lasers or practice of safe laser protocol can be overcome by concomitant use of bench-top simulators such as the Kansai HoLEP simulator which employ real instruments and lasers. 3D printed organ phantoms have been validated for prostate enucleation procedures [15^▪^,^16^] and these also carry such benefits but unlike VR simulators, they inadequately simulate morcellation.

PCNL is another endourological procedure for which simulators have been validated. One study used participant ratings and performance data to compare three simulators [17]; a porcine tissue simulator, the VR ‘PercMentor’ simulator, and a novel immersive VR ‘Marion K181’ simulator. While the immersive VR simulator achieved higher ratings across all metrics than the conventional VR simulator, it did not differ from the porcine model in any domain with participants scoring both similar ratings for simulation fidelity and tactile feedback. The only advantage reported of the immersive VR simulator over the physical porcine model was the absence of radiation exposure.

## LAPAROSCOPIC AND ROBOTIC PROCEDURES

Although cadavers are considered the gold-standard simulation modality, artificial perfusion of cadavers is limited by high cost, positive viral profiles and difficulty clearing the vascular tree. One low-cost, nonbiohazardous physical model for robot-assisted partial nephrectomy (RAPN) [18] directly addresses shortcomings of typical physical simulation with the unique ability to use diathermy (as a grounding pad is included in the model) and thereby practice haemostasis. The model also features customisable pathology in addition to simulation of bleeding and damage to surrounding organs. For laparoscopic partial nephrectomy, a porcine model modified to simulate haemorrhaging [19^▪^] has been developed, with the ability for the extent of bleeding to be adjusted according to trainee progression. The simulation was incorporated into an immersive multidisciplinary scenario in order to aid the development of nontechnical skills alongside the technical operative skills.

Live animal models such as porcine models pose ethical challenges not associated with other simulators. One study [20] aimed to assess trainee perceptions of a porcine model for laparoscopic surgical simulation with key conclusions including the ability to achieve technical progress in emergency surgical scenarios with the majority of participants feeling there is no satisfactory substitute system. A minority of participants considered it unethical to practice the first technical procedures on live animals although such simulation serves to spare patients from risks associated with operating in the early stages of the learning curve.

VR simulation performance for robot-assisted radical prostatectomy (RARP) has crucially been shown to correlate with live surgical performance in the real operative environment thereby increasing its validity as a training modality. One study [21] of 20 surgeons (14 of whom were experts) demonstrated a statistically significant correlation between Mimic Flex VR needle driving scores and contingency recovery at 24 months after real RARP cases, with needle driving scores on the simulator correlating with live operative needle driving scores. Another study reported similar findings [22] noting a positive association for expert surgeons for VR needle hold angle and driving smoothness skills and continence recovery at 3 months. Given that such technical skills influence postoperative outcomes, the findings point towards VR being not only a training tool but also a key assessor of technical performance.

With VR, simulation is moving beyond just basic isolated skills training to full procedural simulation. This has been demonstrated for RARP [23] with superior performance on a FFC being noted for trainees who underwent procedural training over those who only underwent basic VR training or no training at all. Similarly, a canine cadaver model [24] demonstrated face validity for procedural simulation of RARP, particularly for the nerve preservation, vesicourethral anastomosis and lymph node dissection steps. One comparative paper [24] sought to compare skill transferability between VR and dry laboratory simulation environments using vesico-urethral anastomosis tasks. The performance metrics used exhibited moderate to strong correlations between the two modalities, thereby indicating skill transferability. Furthermore, the study authors measured the study participants’ cognitive workload using eye-tracking software, concluding that trainees experienced higher cognitive workloads in both VR and physical simulation than experts. The authors also reported that VR-generated performance metrics are inferior to the dry lab's automated performance metrics in terms of distinguishing expertise, a central aspect of construct validity.

For laparoscopic pyeloplasty, various physical simulators exist with advantages over VR simulators such as significantly lower cost and easy access whilst maintaining anatomical realism. As with aforementioned procedures, 3D printed models for pyeloplasty have been produced [25] demonstrating construct validity with expert laparoscopic surgeons undertaking the procedure in a significantly shorter time than novice participants. Face-validity comparable with more expensive physical simulators has been demonstrated for a low-cost balloon model of laparoscopic pyeloplasty [26] carrying advantages over animal models with decreased risk of infection transmission, ease of access and reusability. Low-cost animal models do have a useful role in robot-assisted surgical simulation such as $10 chicken crop models for robot-assisted pyeloplasty [27] with reasonable procedural realism but unlike VR simulators, they require a robotic system and the costs associated with running this.

## OTHER PROCEDURES

For the management of urological emergencies such as priapism and testicular torsion, there is an absence of validated VR simulators to develop the skills necessary for the therapeutic interventions these conditions require. There is therefore a reliance on physical simulators to facilitate skill acquisition and these have been incorporated effectively into training curricula. For example, a urological emergency simulation curriculum was devised for military surgeons [28] utilising low-cost, lightweight physical simulators to perform procedures including dorsal slit, scrotal exploration, orchiectomy and suprapubic catheterisation. The use of these simulators alongside a didactic teaching session resulted in statistically significant improvements in participants’ performances. Suprapubic catheterisation (SPC) is a procedure for which there has historically been a dearth of reliable, low-cost simulators but in recent years, various models have been designed to address this. One such simulator includes an ultrasound-compatible SPC model [29] which has achieved face and content validity as well as carrying the benefits of affordability and reusability. As discussed in the previous sections, bench models are poor at simulating bleeding compared to live animal and VR simulators but in veterinary surgery, a perfused physical simulator has been developed to practise orchiectomy-related vascular ligation skills [30]. The model utilises a pressurised syringe to simulate perfusion pressure thereby enabling the assessment of trainee performance based on the extent of ‘bleeding’ without reliance on costly VR simulators or ethically-questionable animal models to train this skill.

For penile fracture repair, the first validated simulation model was recently created [31], based on a physical circumcision model adapted with the use of incisions and a red jelly tablet simulating blood clot. Regarding priapism management, one novel corporal aspiration model based on a modified catheterisation teaching model [32] has exhibited a high degree of realism particularly with regard to the simulator's appearance and the tactile feedback it provides. Another low-cost priapism simulator [33] achieved similar positive ratings for face validity and had the significant benefit of reusability with the trainer ‘self-sealing’ after each aspiration and consisting of easily replaceable Penrose drains. Limitations included an undersized replication of the corpus cavernosum which impeded aspiration attempts as well as lacking the attribute of simulating a dorsal penile block.

Reconstructive urology is another field in which validated VR training is absent. Instead high-fidelity, 3D-printed penile models have been produced for the simulation of plaque incision and graft (PIG) as indicated for the treatment of Peyronie's disease [34], achieving high ratings for content validity, face validity and overall experience scores. The absence of VR simulators in the field may be explained by the effectiveness of these cheaper, existing physical simulators but also, as suggested in this review [35^▪^], by the lack of VR's feedback regarding the quality of functional and performance outcomes. The authors also cite the considerable challenge of incorporating the plethora of reconstructive techniques utilised for the management of each condition as another obstacle to the development of VR simulators in the area.

## CONCLUSION

To conclude, physical models are generally superior with regard to cost-effectiveness, the provision of haptic feedback and ease of access, while VR simulators are self-sufficient, reusable and avoid the ethical and infection concerns associated with animal models. Although cadavers have long been considered the gold-standard simulation modality, these may soon be superseded by the advent of highly realistic, perfused 3D printed models capable of procedural simulation incorporating anatomical variation and intraoperative complications [36] at a substantially lower cost. There is a paucity of VR simulators in areas such as emergency urology and reconstructive urology, although existing physical models appear to be adequate in addressing the educational needs of trainees. For the other urological fields, concomitant use of VR and physical simulators is beneficial given the skill transferability [24] and additive effect they have [11]. The type of simulator to be used is also dependent upon whether full procedural simulation or task-based simulation is required, with VR and cadavers generally being the better options for the former and with ‘dry-lab’ bench models being more cost-effective for the latter. In order for more definitive conclusions to be made, further randomised-controlled trials are required to directly compare the educational value of VR simulators versus physical models in a procedure-specific fashion.

## Acknowledgements

*The authors received no source of funding and have no conflicts of interest.*

### Financial support and sponsorship

*None.*

### Conflicts of interest

*Baldev Chahal, Abdullatif Aydin and Kamran Ahmed have no conflicts of interest or financial ties to disclose.*

## REFERENCES AND RECOMMENDED READING

Papers of particular interest, published within the annual period of review, have been highlighted as:

▪ of special interest
▪▪ of outstanding interest
